# MaizeDIG: Maize Database of Images and Genomes

**DOI:** 10.3389/fpls.2019.01050

**Published:** 2019-08-28

**Authors:** Kyoung Tak Cho, John L. Portwood, Jack M. Gardiner, Lisa C. Harper, Carolyn J. Lawrence-Dill, Iddo Friedberg, Carson M. Andorf

**Affiliations:** ^1^Department of Computer Science, Iowa State University, Ames, IA, United States; ^2^USDA-ARS Corn Insects and Crop Genetics Research Unit, Iowa State University, Ames, IA, United States; ^3^Division of Animal Sciences, University of Missouri, Columbia, MO, United States; ^4^Department of Genetics, Development and Cell Biology, Iowa State University, Ames, IA, United States; ^5^Department of Agronomy, Iowa State University, Ames, IA, United States; ^6^Bioinformatics and Computational Biology Graduate Program, Iowa State University, Ames, IA, United States; ^7^Department of Veterinary Microbiology and Preventive Medicine, Iowa State University, Ames, IA, United States

**Keywords:** MaizeDIG, BioDIG, phenotype, genes, QTL, gene model, MaizeGDB

## Abstract

**Background:** An organism can be described by its observable features (phenotypes) and the genes and genomic information (genotypes) that cause these phenotypes. For many decades, researchers have tried to find relationships between genotypes and phenotypes, and great strides have been made. However, improved methods and tools for discovering and visualizing these phenotypic relationships are still needed. The maize genetics and genomics database (MaizeGDB, www.maizegdb.org) provides an array of useful resources for diverse data types including thousands of images related to mutant phenotypes in *Zea mays* ssp. *mays* (maize). To integrate mutant phenotype images with genomics information, we implemented and enhanced the web-based software package BioDIG (Biological Database of Images and Genomes).

**Findings:** We developed a genotype-phenotype database for maize called MaizeDIG. MaizeDIG has several enhancements over the original BioDIG package. MaizeDIG, which supports multiple reference genome assemblies, is seamlessly integrated with genome browsers to accommodate custom tracks showing tagged mutant phenotypes images in their genomic context and allows for custom tagging of images to highlight the phenotype. This is accomplished through an updated interface allowing users to create image-to-gene links and is accessible *via* the image search tool.

**Conclusions:** We have created a user-friendly and extensible web-based resource called MaizeDIG. MaizeDIG is preloaded with 2,396 images that are available on genome browsers for 10 different maize reference genomes. Approximately 90 images of classically defined maize genes have been manually annotated. MaizeDIG is available at http://maizedig.maizegdb.org/. The code is free and open source and can be found at https://github.com/Maize-Genetics-and-Genomics-Database/maizedig.

## Introduction

One of the fundamental relationships in biology is how observable physical and biochemical characteristics (phenotypes) relate to the underlying combination of alleles of the genes within an organism (genotype). Maize is a good case study for exploring this relationship because maize is both agronomically important as the top production grain crop (http://faostat.fao.org/) and serves as a model species. Maize has numerous reference quality genome assemblies available for exploring the genotype to phenotype relationship (Schnable et al., 2009; Lu et al., 2015; Hirsch et al., 2016; Jiao et al., 2017; Sun et al., 2018; Springer and Anderson, 2018), and a great deal of research has been carried out in maize that links mutant phenotypes to genes or regions within the genome, for example, quantitative trait locus (QTL) (Schnable and Freeling, 2011). Other linkages from genetics and genomics data to phenotype include the identification of numerous QTLs and associations of single-nucleotide polymorphisms with phenotypes and traits through genome-wide association studies (Xiao et al., 2017). Many loci and traits are further annotated with accessible high-resolution images. MaizeGDB has more than 2,700 images mapped to genes and more than 1,000 mapped to a gene model with specific genomic coordinates available. In addition, there are an additional 200 images where the specific phenotype feature has been tagged within the image. Most of these images were donated by Dr. Gerald Neuffer, University of Missouri-Columbia. His research created thousands of mutations by treating maize pollen with the known mutagen ethyl methanesulfonate. Many of these were imaged and shared in the book *The Mutants of Maize* (Neuffer et al., 1968) and/or are available at the “Guide to Maize Mutant Phenotypes” website hosted at MaizeGDB (http://mutants.maizegdb.org/). This collection of digital images is under active curation.

Although many of these images are linked to a gene locus, prior to the efforts described here, there was no method to visualize these images within their genomic context *via* the MaizeGDB Genome Browser. This current situation hinders researchers working to prioritize candidate genes underlying traits of interest within a chromosomal region from being able to narrow their search for the causal gene in a quick and easy way.

The first maize genome sequence assembly was completed in 2009 (Schnable et al., 2009). The most recent version of the reference assembly, B73 RefGen_v4, is based on PacBio sequencing and high-resolution optical mapping with an N50 value of 1.18 Mb (Jiao et al., 2017). It also has nearly 40,000 structurally annotated protein-coding genes. In addition, the rapidly decreasing cost of genome sequencing and assembly has led to the availability of a number of new high-quality maize genome assemblies from different maize lines. This suggests that use of a single reference genome may no longer continue to best serve the needs of the maize research community. In addition to B73, reference quality maize genome assemblies and annotations released in the past few years include CML247 (Lu et al., 2015), PH207 (Hirsch et al., 2016), Mo17 (Yang et al., 2017; Sun et al., 2018), European Flints EP1 and F7 (Unterseer et al., 2017), and W22 (Springer et al., 2018). In the near future, we anticipate the release of genome assemblies for dozens, if not hundreds, of *Zea* lines. The high level of phenotypic and genomic diversity within maize—any two given maize lines can be as different as humans and chimpanzees (Buckler et al., 2006)—means these additional genome assemblies will have great value.

Currently, there is a pressing need to better integrate phenomic data within the context of multiple genome assemblies. The current methodology of associating images to genomic data at MaizeGDB is relatively complex: Each gene can have multiple alleles (denoted as variations at MaizeGDB); each image is linked to its appropriate allele; a gene can be linked to a gene model with genomic coordinates. A tool is needed to make simplified and more direct connections between the phenotype and genotype, which in turn will allow researchers to explore a genome or gene region simultaneously in both a genomic and phenomic context. There currently exist tools and resources to help manage, curate, and analyze images such as BisQue at CyVerse (Kvilekval et al., 2010) and BioDIG at GMOD (Oberlin et al., 2013). BisQue is a cloud-based platform that provides support to organize images, integrate metadata, and build complex analyses with the images/metadata. BioDIG is a stand-alone software to integrate images with genomic data. To address the need to curate phenotype images, link to genomic data, and visualize the relationships within the context of a model organism database, we chose to implement an enhanced version of BioDIG.

In this article, we present an open-source, web-based software called MaizeDIG (SciCrunch.org tool reference ID: SCR_016987), a multiple maize reference genome implementation of BioDIG, with enhancements that enable tagging and linkages between images and genes/gene models or QTLs, support for multiple reference assemblies, creation of custom autogenerated genome browser tracks, and expanded search capabilities.

### MaizeGDB

The Maize Genetics and Genomics Database (MaizeGDB; http://www.maizegdb.org) is the model organism database for maize (Portwood et al., 2019). MaizeGDB’s overall aim is to provide a single point of access to maize research data and tools for integration, visualization, and discovery. Stored at MaizeGDB is comprehensive information on genes, alleles, mutants, stocks, molecular markers, gene product information, phenotypic images and descriptions, metabolic pathway information, pedigree information, reference data, sequences including multiple genome assemblies and annotation sets, contact information for maize researchers, and more. Curation of high-quality and high-impact datasets has been the foundation of the MaizeGDB project since its inception over 25 years ago (Schaeffer et al., 2011; Andorf et al., 2016; Harper et al., 2016). Since the first maize genome sequence assembly was announced in 2009 (Schnable et al., 2009), MaizeGDB has provided access to genome assemblies and annotations through GBrowse-based genome browsers and other tools (Sen et al., 2009). Currently, MaizeGDB supports over 10 independent maize genome sequence assemblies of different maize lines, each with their own annotation set(s). A comparison of these genome assemblies reveals a high level of sequence diversity between maize lines, allowing maize researchers to investigate what genotypic combination of alleles leads to the great variety of phenotypes in maize, especially agriculturally important phenotypes such as grain yield and planting density. Genotype-to-phenotype hypotheses can be facilitated by genome visualization tools. One way to integrate high-quality phenotypic data with a reference genome assembly is to link images of phenotypes to genes that have known physical locations on the genome assembly. MaizeGDB currently hosts 4,367 unique mutant phenotype images, of which 2,396 are linked to a locus on the genome. Detailed descriptions of specific phenotypic features within an image can provide additional clarity. To address these needs, MaizeGDB has adapted the BioDIG system to create a maize-specific database of images and genomes.

### BioDIG

BioDIG is a web-based biological database for linking images to genomic data (Oberlin et al., 2013). To link an image to a gene, an image tagging tool called MaizeDIG Workbench is provided. In the workbench, a user can outline and highlight regions within the image, called a “tag” and create links from the tag to genomic coordinates (gene links). BioDIG is designed to work generally for all types of images, but the tagging feature is particularly useful for phenotypes in complex images or where the exact phenotype may be difficult to identify. To handle genomic data, the Generic Model Organism Database (GMOD) Chado database schema is used because it is compatible with standard data formats (e.g., Generic Feature Format or GFF files), and it is easily extensible to add features to genes. The front-end (client-side) user interface is based on the Django framework (https://www.djangoproject.com/) and jQuery.

BioDIG was designed for general organisms, and it needs genome and image data of the target organism. BioDIG provides a tool to build and update genome data with a GFF. However, it requires extra database installations and configurations. We extended BioDIG to use genome and image data from MaizeGDB directly, and it can reduce potential errors or overheads related to the handling of genome and image data because MaizeGDB has well-curated genome data and images for maize. In addition, BioDIG’s functionality was extended by adding novel features such as multiple genome support, enhanced searching, and images shown in dynamic genome browser tracks.

### Connecting Genes to Phenotype Through Gene Links

A large amount of both genomics and phenomics data is available for maize with many methodologies, applications, and tools being utilized. Even with these valuable tools in place, it is still a challenge to visualize and discover relationships between phenotype and genotype. In MaizeDIG, we have defined the relationship between a gene and its phenotype as a gene link. Gene links can be used to give a quick visual representation of the phenotype in any context in which a gene is presented at MaizeGDB. The gene link comprised an image tag and a gene. A single image can have multiple phenotypic features, and each of those features may be represented by a single or multiple genes. Likewise, a mutant allele of a gene can display multiple phenotypes shown in different images. The exact phenotypic features within an image can be annotated with the tagging tool in which a curator can highlight the section of the image by drawing either a rectangular region or freestyle region, and these tagged regions can be linked to a gene(s). These tagged regions along with their gene links represent a map of the phenotype-genotype relationship that can be used in the framework of the MaizeGDB database to address a variety of situations that require an understanding of a gene and the corresponding phenotypes of its alleles. MaizeGDB gene pages also provide functional annotations that are linked to the image. These annotations include Plant Ontology, Gene Ontology, and other ontologies terms, their expression, and descriptions of the mutant phenotypes.

MaizeDIG allows a novel way to visualize these relationships as a track on the MaizeGDB Genome Browser. The MaizeGDB Genome Browser lets a user browse the genomic DNA sequence at varying levels of complexity to see aligned biological features within the context of that genomic region. The MaizeDIG track allows a user to browse a chromosomal region and see images of tagged mutant phenotypes for genes of interest. Some applications of using this track include determining gene function, chromosome walking to genes, identifying enriched biological processes or metabolic pathways, and identifying regulatory regions adjacent to genes.

## Materials and Methods

**Figure 1:**
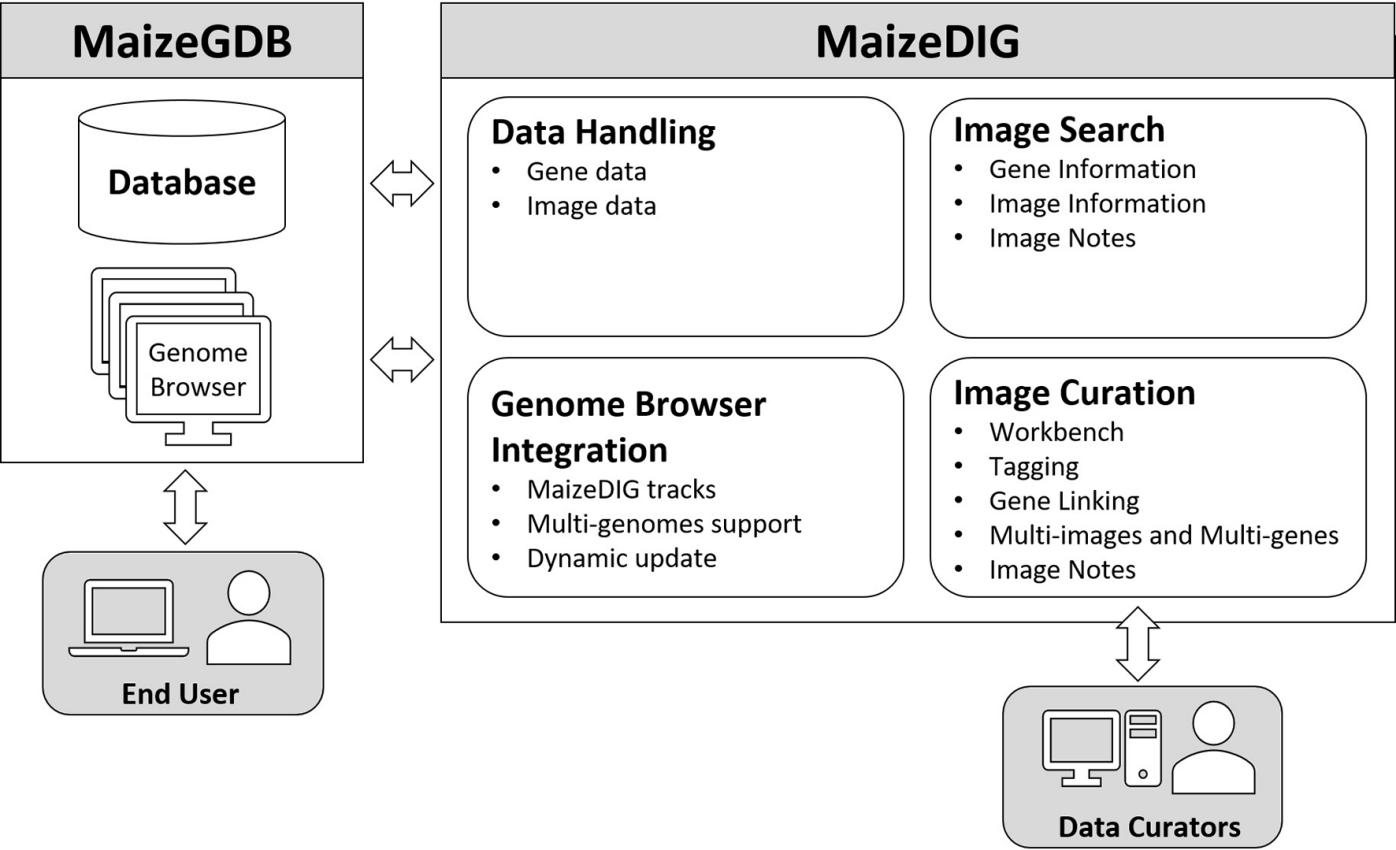
Structure of MaizeDIG. MaizeDIG’s structure is divided into four major components: data handling, image curation, image search, and genome browser integration.

MaizeDIG is a genotype-phenotype linking database based on BioDIG that has been enhanced to show phenotype images on a genome browser and handle multiple maize genome assemblies. MaizeGDB has over 2,700 mutant phenotype images, and many of these digital images are linked to a gene model. MaizeDIG has a set of web-based tools that allows searching, tagging, annotating, and linking images with genes, gene models, and alleles. In addition, MaizeDIG has been integrated with several MaizeGDB Genome Browsers simultaneously. Once an image is tagged to a gene, it becomes available as a custom track for any maize genome assembly that has a gene model associated to that genomic region. There are four functional aspect of MaizeDIG: data handling, image curation, image search, and genome browser integration. Each of these aspects has been enhanced relative to its original implementation of BioDIG. **Figure 1** shows the four main modules, and we discuss details of these in the following subsections.

### Data Process

The raw data used to build the MaizeDIG database are images and genomic mapped loci (e.g., genes). The general data process for MaizeDIG is to load an image, tag the location that best identifies a phenotype, link the tag to a gene or QTL, and provide additional metadata. To make a link between gene and image, both image and genomic data must exist that can be linked together.

### Image Data

All phenotype images at MaizeGDB have been provided by individual researchers. To date, the photographs in MaizeDIG have been different than ones in publication to keep from copyright law violation. Given more recent Open Access licensing, we anticipate the use of images directly from publications with the appropriate Creative Commons license. MaizeGDB has 2,396 images that are each linked to the gene representing multiple alleles that display the imaged phenotype. Most images are of phenotypes caused by mutations in single genes. Images are loaded through the MaizeGDB curation tools, but in the absence of a model organism database, BioDIG can be used as the primary way of uploading images.

### Genomic Data

MaizeGDB acts as the steward of maize genome assemblies. Currently, MaizeGDB hosts genome assemblies for over 10 different maize inbred lines. In the absence of software that can display multiple genomes at the same time, MaizeGDB provides a genome browser for each genome assembly. Each assembly has its own set(s) of structural and functional gene annotations, that is, gene model sets. Classically defined genes are associated to these gene model sets. Information linked to a gene is therefore inherited by the associated gene models sets.

The next sections describe the individual components that make up the process of building a maize genotypic-phenotypic database and pipeline to integrate gene, gene model, and phenotype image data from the MaizeGDB database.

### Gene Data

**Figure 2:**
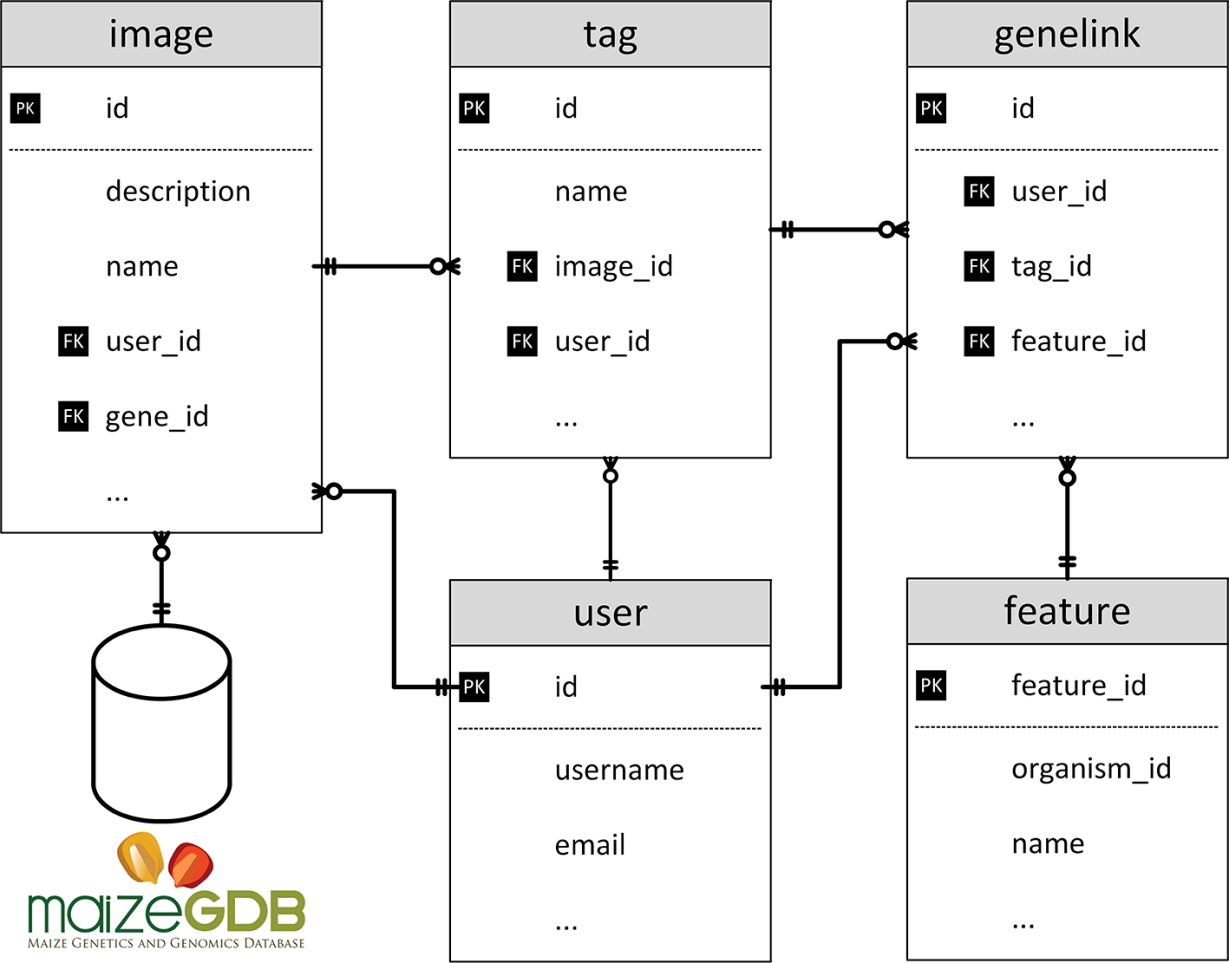
Database structure of MaizeDIG. This image shows the database schema for the five major database tables used in MaizeDIG.

MaizeDIG is an extension of Chado (Mungall and Emmert, 2007), one of the most popular relational database schema for biological research. **Figure 2** shows the table schemas related to genotypic and phenotypic data. The user table deals with all user information including administration accounts. The feature table has the genome data needed from maize. The genelink table deals with linking information between the tag and feature. A full description of the image, genome, and user management modules and details on the underlying BioDIG database are described at GMOD and the original BioDIG article (Oberlin et al., 2013). Although MaizeDIG provides a system to manually link genes to an image, the majority of these associates are preloaded based on information at MaizeGDB. In the MaizeGDB database, images and phenotype descriptors are (independently) linked to alleles, alleles are linked to genes, genes are linked to gene models, and gene models are linked to genomic coordinates. Scripts have been developed to load these associations into the MaizeDIG database. The details of linking between image and gene are discussed in Image Curation section.

### Image Data

To build an initial phenotype image database, 2,721 images with gene information were imported from MaizeGDB to MaizeDIG. These images are based on interesting maize mutations that were photographed, and extensive efforts were taken to map to specific genomic locations using whatever chromosome mapping technologies were available at the time. Indeed, this collection of new maize mutations launched many careers in maize genetics which contributed to diverse areas of plant science such as developmental biology, chromosome mechanics, hormone metabolism, photosynthesis, and more.

The images are mapped to gene(s) in MaizeGDB; hence, each image imported from MaizeGDB has an associated gene called primary gene. An image can have only one primary gene in MaizeDIG. The primary gene does not automatically become a gene link directly, but it functions as the recommended gene. In general, curators use the recommended gene to make a gene link, but it is also possible to choose a different gene(s) as well. There are examples where the same image may exist multiple times in the database with different genes assigned to it. MaizeDIG handles these redundancies by creating image groups, which internally recognizes redundancies and consolidates any gene or gene link data. In addition to gene data, if a caption is available for an image, it is loaded as a description in MaizeDIG.

### Image Curation

MaizeDIG provides an image curation tool suite enabling tagging of phenotypic features, image-gene linking, visualization of image-gene data, and image searching. In this section, we provide details on each of these features.

#### Workbench

**Figure 3:**
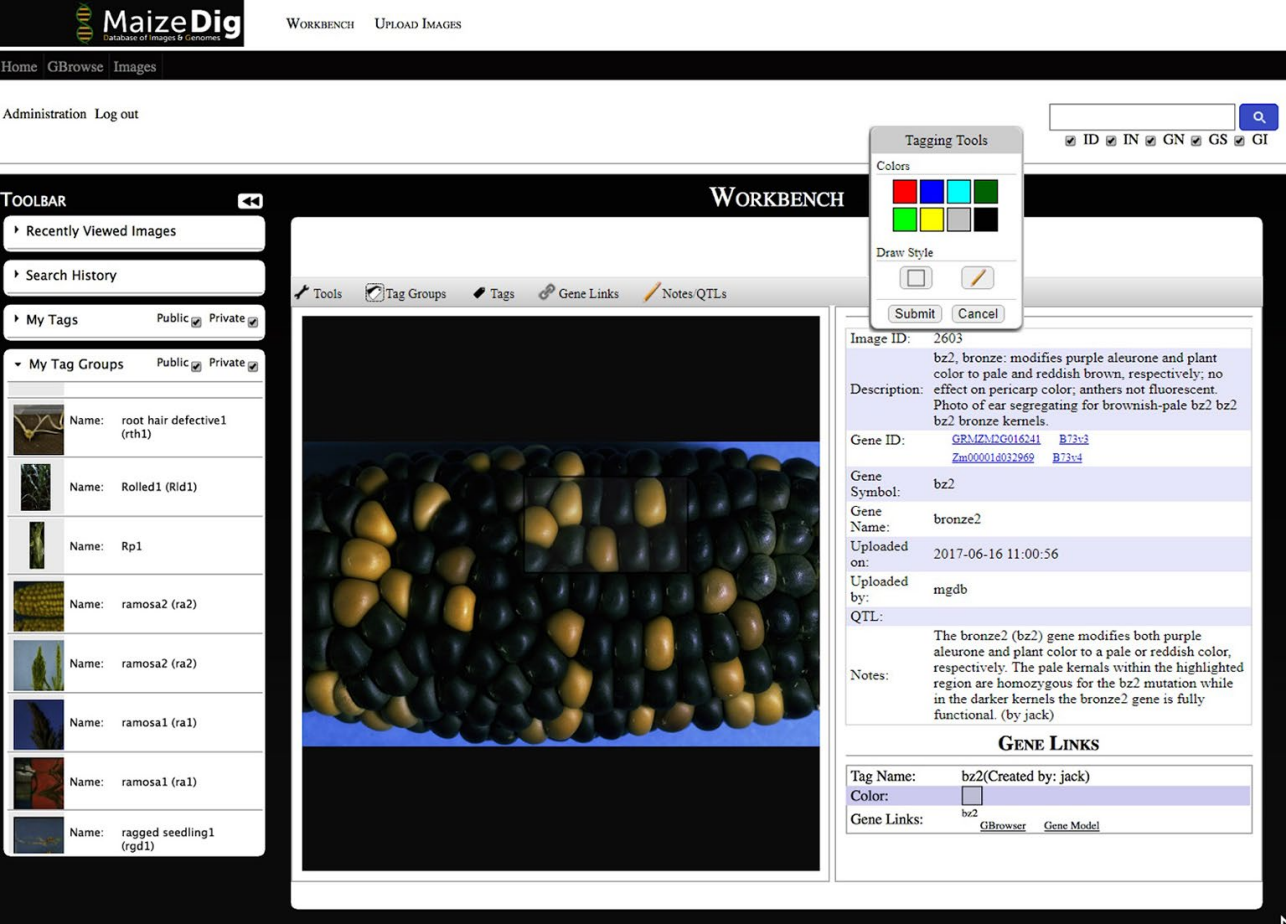
Maize curators workbench. A screenshot of the MaizeDIG Curators Workbench is shown with and image displaying the bronze2 (bz2) kernel phenotype segregating on a maize ear. Tools for image tagging, curation, and linking to the MaizeGDB Genome Browser can be accessed on the toolbar directly above the image. Mousing over each of the five tools activates a pull-down menu with a variety of options. The “Tagging Tools” pop-up that allows a user to add a tag to an existing tag group is shown. The collapsible vertical tool bar to the left of the image allows MaizeGDB curators to review their search history and recently viewed images as well their tags and tag groups. In the upper right-hand corner is a search box that allows curators to search over 2,300 images by Image Description (ID), Image Notes (IN), Gene Name (GN), Gene Symbol (GS), and Gene ID (GI). To the right of the image is the preloaded image description, Gene ID, Symbol, and Name as well as the Image ID. The phenotypic description as it will appear on the genome browser pop-up (minus the curator attribution) appears in the Notes section. The Gene Links information appears at the bottom once a successful gene link has been made between an image tag on the workbench and the genome browser.

Image curation is done within the MaizeDIG Workbench (see **Figure 3**). The workbench is accessible only when a user is logged into his/her account. This allows the curation process to be saved and associated to a user/curator. The workbench is divided into three subsections: toolbar menu (left), image viewing/editing section (center), and detail information section (right). The recently viewed images menu on the left-side toolbar menu is useful for keeping track of the 10 most recently viewed images. The image viewing/editing section has a menu bar on the top. The menu bar includes tags, tag groups, gene linking, and user note features. Additional information such as Image Description, Gene ID, Gene Symbol, Gene Name, Image ID, and User Notes is shown on the right side of the image. The gene ID/symbol/name are associated to a gene, but they do not function as a gene link until activating the gene link using the “Gene Link- > Add New Link to Tag” tool. The image viewing/editing box can show all tags belonging to the image, and even though each tag has an ownership with an associated “user_id” (**Figure 2**), all tags are displayed on the image.

#### Tagging

An image can represent a phenotype by the image itself without additional modification, but in many cases, the phenotypic image will benefit from additional visual annotation to provide clarity. The tagging process specifies a certain region within an image and labeling it with a tag ID. **Figure 3** shows a custom tagging on image. The tagging tool provides eight colors and two drawing styles (rectangular and free pen). Multiple tags can be created on a single image.

#### Gene Linking

Once an image has been tagged, a gene link between the tag and a gene can be created by adding a gene to the tag. The “Add New Link to Tag” tool provides a way to complete this task, which is called creating a “genelink.” A genelink is required to have a one-to-one relationship between a tag and a gene. A gene symbol or gene model can be assigned to a tag, and typically, a single image is linked to a single gene. It is also possible that a single image can show multiple phenotypes or have phenotypes associated to multiple genes and therefore need to be mapped to the multiple genes. The gene link allows connecting to MaizeGDB database such as Genome Browser and Gene model page.

#### Linking to QTL Data

Most phenotype images at MaizeGDB are for monogenic phenotypes. MaizeGDB does contain more complex phenotypes and traits that have been associated to multiple loci across the genotype. These QTLs are also supported in MaizeDIG. Within the MaizeDIG Workbench, a QTL ID can be added to any image. Similar to a gene link, the QTL ID connects the image to QTL information in the MaizeGDB database.

#### Many-to-Many Relationships Between Images and Genes

In the phenotypic data handling section, we discussed the relationship of images and genes within the context of two different scenarios: multiple images are associated to a single gene, and multiple genes are linked to a single image. MaizeDIG has addresses these two scenarios with the “Image Group” tool. In the “multi-image to single-gene” case, it is straightforward for the gene to be assigned to multiple images. However, additional treatment is needed for the “multi-genes to single-image” situation. In this case, the image data are duplicated with different “Image ID” for each primary gene, but is still considered a single image within the “Image Group” tool. This ensures that all tags and gene links in the same group appear in each image.

#### Image Notes

Image notes provide another mechanism for communicating additional information in the image curation process. It is important to be able to share opinions/comments among curators. Curators can create/edit image notes regardless of its ownership. In addition, images can be queried by the image notes using the image search tool.

## Enhancements

### Image Search

MaizeDIG offers an image search tool that has been enhanced relative to BioDIG as follows. A curated image has detailed information such as image description, gene model IDs, gene symbol, gene name, and image notes. Any of these categories can be searched individually or together using the image search box. With thousands of images loaded into MaizeDIG, the flexible search tool is needed to locate the correct image to curate. In addition, an image search history has been implemented in MaizeDIG that saves 10 most recent searched images and the search settings in an expandable box on the left side of the workbench.

### Downloading Images and Metadata

**Figure 4:**
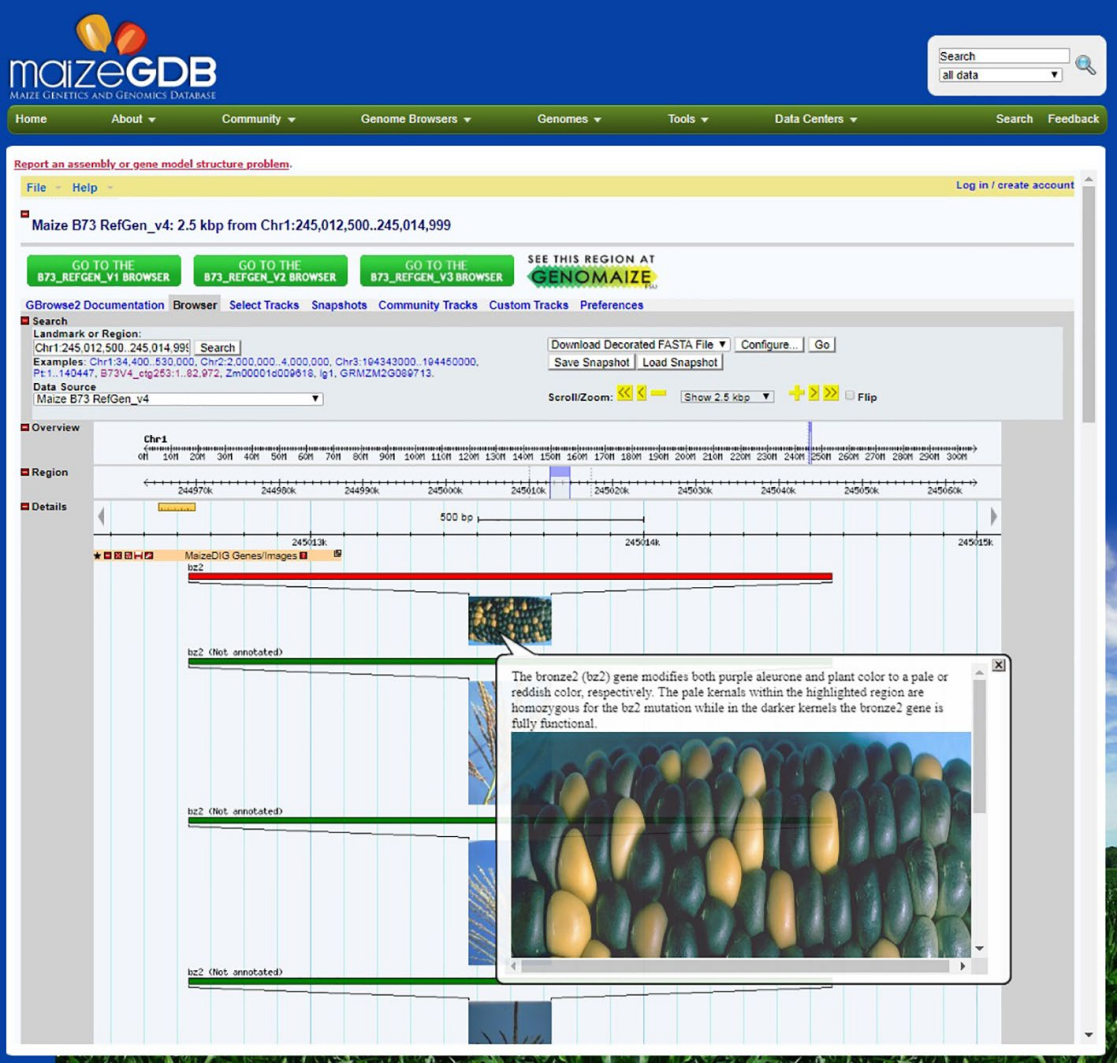
MaizeDIG on the MaizeGDB Genome Browser. A screenshot of the MaizeDIG curated bronze2 (bz2) image and how it presents in the context of the MaizeGDB Genome Browser is shown. Mousing over any of the MaizeDIG images activates an image pop-up, while clicking on the image takes you to a webpage with a full screen image and phenotypic description. The majority of images in MaizeDIG are high-resolution and present well as a full screen image. Manually curated images have a red banner and are automatically sorted to the top of the image stock, while unannotated images have a green banner and are shown below. Note the multiple images shown for bz2. Some phenotypes may only have one image, while other phenotypes may have ten or more.

Images and associated metadata can be downloaded from either the workbench or an image record page. The “Tools|Download Image Data” menu item will open up a simple download form where a user can select the image attributes and data format. The attributes include URL of image, upload data/user, image tags, image file, tag groups, and gene links. The image tag data will include the coordinates and color options of the highlighted regions. The options for the output format are JSON and XML. After selecting the attributes and format, a zip file will be downloaded that includes the image and attribute file. Genome Browser integration MaizeGDB’s GBrowse (Donlin, 2009) instance provides hundreds of tracks across multiple genome assemblies, but up until now did not include any phenotypic data. The genome browser is one of MaizeGDB’s most accessed resources, receiving nearly 80,000 unique page views in 2018 according to usage statistics from Google Analytics, and so adding genotype-phenotype relations to this resource raises the accessibility and usability of these data. Therefore, we have created dynamically loaded tracks that contain all of the images, tags, gene links, and user notes in MaizeDIG. The tracks are available for all of our genome browsers that have gene model associations with the reference genome (B73) which as of this publication includes the following: B73v3, B73v4, W22v2, PH207, Mo17 (x2), B104, EP1, F7, and Zx-PI566673. As new genomes are incorporated into MaizeGDB, new MaizeDIG tracks will also be created for their respective genome browsers. All MaizeDIG tracks are dynamically updated when a user creates (or removes) a gene link to a tag. Upon creating a gene link, a new feature is inserted into the track at the location of that specified gene, and vice versa if the gene link is deleted. The feature shows a thumbnail of the image, and mousing over it reveals the full size of the image along with all of its tags, user notes, and other gene links. **Figure 4** shows an example of this track on the B73v4 genome browser.

## Curation Case Study

After the initial bulk upload of over 2,300 images and metadata, it was both possible and advisable to manually annotate select images that present a clear representation of the relevant phenotype associated with a gene mutation. To accomplish this, MaizeGDB curators added manual annotations for every classically defined gene (Schnable and Freeling, 2011) in the database that were associated with a mutant phenotype image. In total, approximately 90 genes were curated, which enables the end user to unambiguously identify the exact phenotype in both manually curated and noncurated images. The manual curation approach taken by MaizeGDB curators varies somewhat depending on the particular phenotype and its presentation within the image. In ideal situations, the curator is able to deploy a “compare and contrast” approach if the mutant and nonmutant phenotype is present within the same image. However, this is not always possible, and a different curation strategy must be taken in these situations. In **Figure 3**, a screenshot taken from the curators MaizeDIG workbench displays an image of a maize ear, with a majority of kernels having a dark purple color, and interspersed between them are a few bronze-colored kernels. The *bronze2* gene (*bz2*) when fully functional causes the kernels to acquire a dark purple color while the bronze colored kernels represent the *bz2* gene in a nonfunctional or mutated state.

The manual curation process is initiated by the creation of a tag group from the “Tag Groups” pull-down menu directly above the image. From this menu, it is possible to create multiple tag groups and delete or modify them. In practice, it is rare to have multiple tag groups attached to an image, although certainly there are situations where this proves to be a useful feature. Once a tag group has been created and named, a tag can be created from the “Tags” pull down in the menu bar. Once the tag has been activated, a “Tagging Tools” pop-up appears (**Figure 3**) with a choice of both drawing style (freehand or box) and eight tag fill color options. After selecting both the drawing style and color fill option, the curator moves directly to the image and highlights the area of interest using the mouse. In this example, kernels with (bronze) and without (dark purple) the bz2 mutation are selected to be within the tagged area. Once the tagged area has been selected, and the submit button on the “Tagging Tools” pop-up is chosen, an additional “Submit Tag” pop-up appears (not shown in **Figure 3**), and a “Tag Group” can be chosen and a name attached to the new tag. Once an image tag has been created, it is necessary to create a “Gene Link” to make a link or connection between the gene/image on the curation workbench and the gene’s location on the MaizeGDB Genome Browser, which is what the end user will see and interact with (**Figure 4**). This is accomplished by accessing the “Gene Links” pull-down menu directly above the image. If there are multiple tag options, a tag is selected, and the relevant gene symbol (*bz2* in this example) is entered in the “Gene or Locus” field.

The manual curation process is completed by the addition of a phenotypic description in the pop-up from the “Notes” section above the image (**Figure 3**) in the curation workbench. When completed, the phenotypic description appears both on the right-hand side of the image (**Figure 3**) in the workbench and in the image pop-up on the MaizeGDB genome browser (**Figure 4**). It is worth noting that the “Description” is seen only on the workbench view and not in the genome browser pop-up that is visible to the end user. Information in the “Description” section is pulled from MaizeGDB records and often contains information that is not relevant to the phenotype being presented. In general, MaizeGDB curators strive to keep the phenotypic description simple and avoid excessive use technical genetic jargon, while still maintaining a robust scientific description. At the outset of the MaizeDIG project, our goal was to provide simple explanations for the phenotype when presented in the context of the genome browser. For those interested, more detailed explanations can be found in the gene model records at MaizeGDB. MaizeGDB curates maize research papers and will use MaizeDIG to curate phenotype images found in these manuscripts.

## Conclusion

MaizeDIG facilitates a major priority at MaizeGDB which is to make accessible high-quality data and create added value through curation, integration, or through providing tools that visualize and/or analyze the data. This tool enables the integration of phenotype and genotype data in a model species rich in both data types. This is done directly by making dynamic tracks on multiple MaizeGDB Genome Browsers, the most widely used tools at MaizeGDB, for genomic exploration. Having the phenotype images in their genomic context along with other mapped features (e.g., gene models, expression data, polymorphisms, etc.) enables users to prioritize gene candidates for phenotypes of interest. This data will be valuable in forward genetics by providing quick visual clues for prioritizing genes. For example, if a region of the genome has been associated with the phenotype “photosynthetic capacity,” genes could be quickly identified by looking at mutant phenotypes related to leaves. In addition, these images can be used in a variety of settings to prioritize candidate genes identified through coexpression, differential expression, sequence similarity, domain sharing, functional enrichment, and subcellular localization. This represents a major step forward as experimental determination and/or validation are both time-consuming and expensive. MaizeDIG will be a valuable addition to the MaizeGDB manual annotation workflow.

These use cases are not unique to maize, and MaizeDIG’s availability as a free and open-source resource will allow the application of integrating phenomic images and genomic data to other databases and projects.

## Data Availability

The datasets analyzed for this study can be found in MaizeGDB at http://maizedig.maizegdb.org. The source code is free and open source and can be found at (https://github.com/Maize-Genetics-and-Genomics-Database/maizedig).

## Author Contributions

All authors were involved in conceptualization, writing, reviewing, and editing of the manuscript. CL-D, IF, and CA were responsible for Funding Acquisition. CA provided project administration. KC and JP developed the software and data visualization tools. JG and LH provided data curation.

## Funding

This work was supported by the US Department of Agriculture-Agricultural Research Service (project no. 5030-21000-068-00-D) and an award from the Iowa State University Presidential Interdisciplinary Research Initiative to support the D3AI (Data-Driven Discovery for Agricultural Innovation) project (to CL-D, IF, and CA). For more information, see http://www.d3ai.iastate.edu/. IF was funded, in part, by NSF award ABI 1458359. Support to develop the “Guide to Maize Mutant Phenotypes” dataset was derived from the NSF’s ABI funding (DBI 0743804) to CL-D and Gerry Neuffer. The funders had no role in study design, data collection and analysis, decision to publish, or preparation of the manuscript.

## Conflict of Interest Statement

The authors declare that the research was conducted in the absence of any commercial or financial relationships that could be construed as a potential conflict of interest.
